# Perception and adoption of competency-based training by academics in Ghana

**DOI:** 10.1186/s40594-018-0148-x

**Published:** 2018-12-03

**Authors:** Camillus Abawiera Wongnaa, Williams Kwasi Boachie

**Affiliations:** 1 Department of Agricultural Economics, Agribusiness and Extension, Kwame Nkrumah University of Science and Technology, Private Mail Bag, University Post Office, Kumasi, Ghana; 2 Department of Accounting Studies Education, University of Education, Winneba, Kumasi Campus, Kumasi, Ghana

**Keywords:** Competency-based training, Adoption, Perception, Faculty members, Logit model, Likert scale

## Abstract

**Background:**

With the rise in graduate unemployment and the poor linkage between university education and industry, competency-based training (CBT) is gaining popularity in Ghana’s universities as a way of producing business-oriented and well-grounded graduates for industry who are ready to make use of knowledge acquired in university education to establish businesses that will help reduce unemployment in the country as well as working effectively in the nation’s industry and service sectors. With CBT yet to be introduced in most Ghanaian tertiary institutions, information about academics’ perception and willingness to adopt the methodology is crucial. This study examined the perception and adoption of CBT by academics in Ghana using cross-sectional data collected from 300 faculty members of Kwame Nkrumah University of Science and Technology (KNUST) using a structured questionnaire. Descriptive statistics, 5-point Likert scale, perception index, and the logit model were the methods of analysis employed.

**Results:**

The results of the survey showed that the overall perception index was 0.49, indicating that generally faculty members of KNUST agreed and had a positive perception of the potential of CBT in instilling in students employable skills. The logit results also showed that the probability of adoption of CBT is positively influenced by participation in CBT workshops, effective supervision of faculty members by university authorities, availability of teaching aids, and availability of incentives. Conversely, adoption was found to be negatively influenced by teaching load and number of undergraduate students per class.

**Conclusions:**

We conclude that provision of appropriate teaching and learning resources that complement adoption of CBT, incentives, and competency-based education training for academics by university authorities and stakeholders in Ghana’s tertiary education will enhance the adoption of CBT methodologies.

## Background

It is important for job seekers to acquire knowledge, skills, and learning capacities in order to prepare themselves for changing operations in industry as well as labor market conditions. Globally, employers seek highly qualified and skilled employees who comfortably respond to changing, complex needs and trends in contemporary workplaces (Andrews and Higson 2008; Boahin and Hofman 2013). The implication is that, for successful employment, students and would-be employees do not only require technical skills but also they need to obtain employable skills (Gibb 2004). With employable skills, students develop, adapt, and transform their industry skills to new contexts (NCTVET 2006). It is therefore important that lecturers integrate employable skills and technical competencies so as to equip students with skills that will help them participate effectively in a wide range of social settings (Boahin and Hofman 2013).

A key methodology in teaching that emphasizes the development of employable skills is competency-based training (CBT). The urgent need to bridge the gap between academia and industry as well as developing competencies and capabilities instead of qualifications is the focus of CBT reforms worldwide (Keating 2008). According to Wesselink and Wals (2011), contemporary interest in CBT is a reflection of continuous growth in the recognition of the great need for students to be equipped with competencies that will help them in their future career as well as help develop those competencies so that they can adapt to and anticipate future growth and developments of their work. In spite of some features that are peculiar to CBT, many academic fields differ in the design and content of their curricula, delivery, assessment, and the specific employable skills they give to students and prospective employees (Kwok 2005; Yorke 2007). Barrie (2005) stated that a student-centered approach to teaching and learning that reflects background characteristics is required for the growth and development of employable skills. In addition, employable skills can be effectively developed through industry-based learning, internships/attachments, and practices in various communities (Crebert* et al. 2004). Yet, training in most Ghanaian universities usually focuses only on the growth and development of theory to the neglect of employable skills. This implies that employable skills that are demanded by the job market are poorly developed and taught in Ghana’s tertiary institutions (NCTVET 2006). Multiple concerns have been raised about the quality of knowledge, skills, and performance exhibited by young employees (Jossberger et al. 2010) and the increasing gap between the skills of new graduates and the demands of the job market (Andrews and Higson 2008). That is, poor employable skills could be a major cause of high rates of unemployment among university graduates in Ghana (Boateng and Ofori-Sarpong 2002). Over the years, universities have not succeeded in producing skilled and capable personnel due to theory-based curricula, inadequate teaching and learning materials, and inefficient systems of industrial attachment which leads to poor job placement of graduates in the labor market (JICA 2001). Poor employable skills of university graduates force most employers to take would-be employees through longer orientation and probation periods before selecting the best-performing employees (Boateng and Ofori-Sarpong 2002).

In response to the aforementioned challenges, policy makers, through the Council for Technical and Vocational Education and Training (COTVET), have suggested the introduction of CBT into Ghana’s university education to equip graduates with the required employable skills in order to achieve economic competitiveness at the global level, as well as reduce unemployment among the youth (CPTC 2006). With the rise in graduate unemployment and the poor linkage between university education and industry, CBT is gradually gaining popularity in Ghana’s universities as a way of producing business-oriented and well-grounded graduates for industry who are ready to make use of knowledge acquired in university education to establish businesses that will help reduce unemployment in the country as well as working effectively in the nation’s industry and service sectors. In the CBT approach, students study appropriate theories as well as practice required skills needed to operate effectively and efficiently in industries and the public sector as well as create and manage businesses for themselves, making them well-grounded to take up any challenge. The truth is, the government and the private sector are unable to absorb all graduates into the public sector, leaving many of them unemployed. Meanwhile, graduates learn a lot in school which they can take advantage of to create jobs for themselves through establishment of businesses. CBT prepares them to be able to do this efficiently by giving them the requisite skills. That is, CBT approach to teaching in tertiary institutions enhances job accessibility better than the normal lecture approaches and at the same time equips trainees with considerable skills required in industry (Acakpovi and Nutassey 2015). With CBT yet to be introduced in most Ghanaian tertiary institutions, information about academics’ perception and readiness to adopt the methodology is crucial, given that they are the key implementing agents. The current study seeks to analyze the perception and adoption of CBT by faculty by finding answers to the following questions: What is faculty’s perception on CBT methodology? Are faculty members ready to adopt CBT in their teaching activities? What factors influence faculty members’ decision to adopt the CBT methodology? Finding answers to these questions is key to the successful implementation of the methodology in Ghana’s universities as it will make available information needed by stakeholders to devise strategies aimed at encouraging faculty members to adopt CBT in their teaching activities.

## Literature review

### Competency-based training

The term “competence” is an evolving concept and therefore has different meanings for different people and nations, depending on their institutional structures and labor processes (Brockmann et al. 2008; Tonhom et al. 2014). For some authors, competence is defined as the ability to perform particular tasks and roles to the expected standards (Mulder et al. 2007) or the capacity to accomplish the key occupational tasks that characterize a profession to satisfactory standards (Kouwenhoven 2010). Recently, the word competence has also been explained by Haddouchane et al. (2017) as “the ability to do something successfully or efficiently” or “a proven ability in a particular subject area as a result of the amount of knowledge possessed which can be assessed”. These definitions view competence as functional, task-oriented, and industry-focused preparation with which individuals can apply the relevant skills and attitudes in a required workplace environment. Competency-based training (CBT) is an approach that allows students to earn qualifications through demonstration of skills and knowledge in a required subject area using a series of carefully designed assessments. Under this, students take tests, write papers, complete assignments, and undertake industrial attachment. With this model, instead of focusing on credit hours, qualifications are awarded through tangible evidence of learning. Outcomes and assessments are the bookends of CBT (Marguerite 2014; Aboko and Obeng 2015). This is in contrast to the traditional form which places emphasis on theoretical aspects of skills training. It is expected to enhance individual industry-specific needs rather than the group (Albanese et al. 2008; Anane 2013). CBT provides training to meet industry-specific needs rather than individual achievement relative to others in the group. The approach has emerged as a useful tool that could be used to address shortfalls in contemporary approaches to training. Ansah and Enerst (2013) reported that, many countries now employ CBT in response to the needs of new entrants into the world of work. It is also suitable for other learners desirous of upgrading their technical skills for existing jobs. Competency-based training (CBT) is demand-driven, and outcomes are based on standards generated from industry. Such standards form the basis upon which curriculum assessment and learning materials are designed and developed (Marguerite 2014). This approach ensures that all learners gain the requisite knowledge, skills, and attitudes or values to be successful at work. Under the approach, each learner is assessed to find the gap between the skills they need as described in the training module and the skills they already have. The difference between the two is the skills gap. A training program is then developed to help the learner acquire the missing skills. Following this, standards are defined to determine expected occupational roles to be performed in the world of work. These standards could be grouped into units to form the basis of certification or awards for individuals who have successfully undertaken the program. Credit values are assigned to units, and this could be based on the content and notional time needed to complete the process. For trainees to be assessed competent, they need to demonstrate their ability to perform tasks to the standard expected in employment (Aboko and Obeng 2015).

In Ghana, there has been a notable wide-ranging gap in skills acquisition from universities and industry-needed skills (Akudugu 2017). For instance, according to Bawakyillenuo et al. (2013), over 80% of firms who responded to questions on skills gaps indicated that there are a significant number of skills that are lacking in the labor market, but are in short supply by tertiary institutions. The skills improvement as well as hands-on training has the capacity to enhance industrial growth and development. The absence of training in practical and entrepreneurial skills remains a recurrent point of criticism in formal employer feedback from Ghana Employers Association (GEA 2006). To this end, students are attached to industries for at least 1 month as the first step towards inculcating practical knowledge and skills in their area of discipline. This is aimed at addressing the notable deficiencies in the theoretical method of training (Aboko and Obeng 2015). Again, the requisite skills required by industries and those assumed to be acquired from the universities appear to be sub-standards when viewed against the competencies required for performance on-the-job (Obeng et al. 2013). A tracer study conducted by Boahin et al. (2010) reported that, 28% of graduates undertake professional formal training after completing their programs. Also, 33% of the graduates from the business programs undertake further formal training after finishing their study program, while for those from the engineering and applied arts and science and technology programs 25% and 19%, respectively, were involved in further training. Although several government policy initiatives have been implemented, there is still the need to increase the skills level of the workforce in order to support industries to increase productivity (GoG 2014). This, according to Ayariga (2013), would help address unemployment challenges bedeviling the country. It was also reported that inadequate level of skilled labor is making it impossible for manufacturers to be competitive (GoG, 2014). Hence, it is the expectation of all stakeholders that the adoption of CBT would help address the skills gap in university graduates.

### The need for adoption of competency-based education

According to Wu (2013), in the private and public sectors, competency-based training is a popular method that focuses on improving employees’ knowledge, abilities, skills, and organizational performance. Boahin and Hofman (2013) stressed the need for academic disciplines to determine specific employability skills required for social and community practice, as a basis for enhancing the development of employability skills in training programs. Studies on strategies aimed at adopting CBT in tertiary institutions are key because a recent survey of 539 chief academic officers indicated that although 79% were in support of awarding academic credit based on competency, only one half reported that their institution currently engages in CBT (Jaschik and Lederman 2016). CBT may indeed be among a multitude of strategies embraced by higher education aimed at promoting student success and adult degree completion (Burnette 2016). It is the fastest growing model in higher education today and when a competency program is developed correctly, it creates the opportunity for the student to be assessed on the skills and knowledge he or she already has while also concentrating on the skills that he or she needs to develop (Cunningham et al. 2016). In the studies conducted by Woodhouse and Rasar King (2009) as well as Cecil and Krohn (2012), the development of CBT content of a program with effective outcome assessment must follow a series of steps. These include working with stakeholders to develop the program’s goals as they relate to teaching, research, and service, creating program goals that help decode the competencies, synthesizing and listing core and concentration competencies, reviewing a list for gaps and overlaps in competencies by evaluating the course learning outcomes and assessments, developing a quality assurance process using syllabi, evaluating experiential learning and applied research opportunities to develop competencies at a higher level, creating an assessment process, and documenting student learning across the curriculum and continual curriculum improvement based on assessment and evaluation findings. These steps, when followed, make CBT a preferred teaching methodology vis-à-vis traditional teacher-centered approach.

CBT allows students to progress at their own pace and has been well received by a great number of students worldwide (Gravina 2017). Indeed, according to Haddouchane et al. (2017), CBT is characterized by the versatility of the teaching methods on the one hand and, on the other hand, by a wide array of activities that replace the traditional lecture-based courses, such as case studies and scenario-based teaching. This approach is now applied in many corners of the world, including the USA, Australia and Europe, and has become “the brand of the new educational policies supported by the United Nations Educational, Scientific and Cultural Organisation (UNESCO), the Organisation for Economic Co-operation and Development (OECD) and the States involved in the Bologna Process, that is aiming to render the dissemination of knowledge an engine for economic and social development.” Added to this is the role of CBT program in promoting international students’ effective cross-cultural adjustment by strengthening their inner capabilities in key areas such as adaptability/flexibility, well-being, growth, creativity, and innovation (Azevedo et al. 2015). Sule (2015) also indicated that competency-based training and development affects employee job performance since there exists a significant positive relationship between training and development and employee performance. Nash et al. (2012) insisted that trainees aspiring to enter specialty areas of practice in any profession need to acquire both core competencies and focus on advanced levels of competencies associated with their area of specialty practice. It indicated that standards of competence are the foundation of credibility for any profession, including those in health care, education, legal, and governmental service. Although there are some existing problems in the implementation of CBT, according to Prabawati and AOktariyanda (2018), CBT is an ideal form for a human resource development program. Through CBT, employees within an organization can reduce or eliminate the differences between the existing performance and the potential performance that can improve the knowledge, expertise, capabilities, and skills that support the achievement of the vision and mission of the organization. Kiguli-Malwadde et al. (2014) reported that CBT has emerged as an important change to education in Sub-Saharan Africa with schools adopting it as an approach to transformative education. According to the study, Makerere University and the University of Ibadan have successfully adopted CBT and show that CBT can be implemented even for the low-resourced countries in Africa, supported by external investments to address the human resources gap.

## Methodology

### The study area

The study was carried out in the Kwame Nkrumah University of Science and Technology in Ghana. The university is currently organized into the following Colleges: College of Agriculture and National Resources, College of Art and Built Environment, College of Engineering, College of Health Sciences, College of Humanities and Social Sciences, and College of Science.

### Sample size and sampling technique

The study employed the formula advanced by Yamane (1967) to determine sample size:

1 $$ n=\frac{N}{1+N{e}^2} $$

where *n* = desired sample size, *N* = the finite size of the population, *e* = maximum acceptable margin of error as determined by the researcher, and 1 = a theoretical or statistical constant. In the year 2014, the Kwame Nkrumah University of Science and Technology (KNUST) had 943 faculty members comprising 813 males and 130 females (KNUST Basic Statistics 2014). Therefore, with a 5% margin of error, the sample size for the study was calculated as:

$$ n=\frac{943}{1+943{(0.05)}^2}=281 $$

For an even distribution of the sampled faculty members in the various colleges, 19 respondents were added to make up to 300.

Multi-stage sampling technique was employed in this study. In the first stage of the sampling design, all the six (6) colleges of KNUST were purposively selected. In the second stage, two (2) faculties were randomly selected from each college to give a total of 12 faculties. Stage three of the sampling design involved selecting 25 faculty members from each faculty. For a fair representation of each department in the selected faculties in the sample, stratified sampling technique was used to select the respondents in proportion to the number of faculty members of each department. The final stage involved systematically selecting a faculty member from a list of faculty members in the department at random and then selecting every *k*^*th*^ (sampling interval) faculty member in the list or frame. *k* was calculated by dividing the size of the population of the faculty members in a particular department by the sample size.

### Analytical framework

The study employed descriptive statistics in presenting socioeconomic characteristics of the respondents. Basically, frequency tables, mean, and standard deviation were the specific descriptive tools employed.

The Likert scale was also used to analyze a faculty member’s perception on the crusade for implementation of competency-based training in tertiary institutions. The mean score $\widehat{X}$ of a perception statement on the Likert scale is computed as:

2 $$ \widehat{X}=\frac{\sum {f}_{ij}{x}_{ij}}{n} $$

where *x* is the ranked value of a perception statement *i* on the 5-point Likert scale and *f* is the total number of respondents assigning value *x* to a perception statement *i* on the 5-point scale. The 5-point Likert scale takes a ranked value of 1 if respondent *j* strongly agreed to a perception statement *i*, 0.5 if agreed, 0 if respondent is undecided (neutral), − 0.5 if disagreed, and − 1 if strongly disagreed. The parameter *n* is equal to the total number of respondents. The overall perception index (PI), which reflects the general agreement of all respondents on all the perception statements on the Likert scale, is computed as:

3 $$ \mathrm{PI}=\frac{\sum \frac{\sum {f}_{ij}{x}_{ij}}{n}}{\mathrm{Number}\ \mathrm{of}\ \mathrm{Perception}\ \mathrm{Statements}} $$

All variables have their usual meaning.

The framework employed to examine factors influencing adoption of CBT is the adoption behavior model where faculty members respond individually and differently to the innovation. A faculty member may respond fully by using the entire package of CBT or may not respond at all or he/she may respond partially. Those who respond may do so slowly or quickly. This adoption behavior model used for this study is based on the threshold theory of decision-making proposed by Hill and Kau (1973). The theory suggests that anyone who has a choice to make has a reaction threshold, which is influenced by many factors. There is a threshold in the domain of explanatory variables below which the stimulus will cause no appreciable response. A reaction will only occur when the stimulus is strong enough to reach the threshold. Choices of this nature are usually modeled as:

4 $$ {y}_i={\beta}_i{x}_i+{u}_i $$

where *y*_*i*_ is equal to one if CBT is adopted by the faculty member and zero otherwise. That is,

*y*_*i*_ = 1 if *x*_*i*_is greater than or equal to a critical value *x*^∗^ (*y*_*i*_ = 1 if *x*_*i*_ ≥ *x*^∗^) and

*y*_*i*_ = 0 if *x*_*i*_ is less than a critical value *x*^∗^ (*y*_*i*_ = 1 if *x*_*i*_ < *x*^∗^).

*x*^∗^ represents the combined effect of the explanatory variables at the threshold level.

The decision to adopt CBT by a faculty member is dichotomized so a faculty member may choose to either adopt or not adopt as earlier stated. This is a binary choice model involving the estimation of the probability of adoption of CBT, *y* as a function of explanatory variables *x*. For this study, *y*_*i*_ therefore takes a value of 1 if a faculty member adopts CBT and 0 if he/she does not adopt it. The estimation of such qualitative response model allows us to estimate the conditional probability that *y*_*i*_ assumes one of the specified values. Thus, the probability that a given faculty member will adopt CBT *β*_*i*_*x*_*i*_ > *x*^∗^ (*y*_*i*_ = 1) is given as:

5 $$ {P}_i= Prob\left({y}_i=1\right)=F\left({\beta}^{\prime }{x}_i\right) $$

The probability that a faculty member will not adopt CBT is, therefore, given as:

6 $$ 1-{P}_i= Prob\left({y}_i=0\right)=1-F\left({\beta}^{\prime }{x}_i\right) $$

where *y*_*i*_ is the observed response of the *ith* observation of the response variable *y*, and *x*_*i*_ is a set of explanatory variables of the *i*th respondent. The function *F* may take the form of a normal logistic or other probability functions (Degu et al. 2000; Greene 2005).

The logit model was employed to analyze the factors influencing adoption of CBT by faculty members of Kwame Nkrumah University of Science and Technology (KNUST). The model, *F*(*β*^′^*x*_*i*_), uses a logistic cumulative distribution function to estimate *P*_*i*_ (Pindyck and Rubinfeld 1998) and is empirically specified as:

7 $$ y={\beta}_0+{\beta}_i{x}_i+{\varepsilon}_i $$

where *y*= adoption of competency-based training (CBT), measured as a dummy (1 for willingness to adopt CBT and 0 otherwise); *x*_*i*_ are the factors influencing adoption of CBT and include *x*_1_= age of respondent, measured in years; *x*_2_= teaching experience, measured in number of years of teaching at the university; *x*_3_= off-campus activities, measured as a dummy (1 for faculty member engages in off-campus activities and 0 otherwise); *x*_4_= competency-specific training, measured as a dummy (1 for participated in a CBT workshop in a specific field and 0 otherwise); *x*_5_= participation in CBT workshop, measured as a dummy (1 for participated in CBT workshop before and 0 otherwise); *x*_6_= effective supervision, measured as a dummy (1 for effective supervision of teaching activities by university authorities and 0 otherwise); *x*_7_=availability of teaching aids, measured as a dummy (1 for availability of CBT complementary teaching aids and 0 otherwise); *x*_8_=availability of incentives, measured as a dummy (1 for provision of incentives for adoption of CBT by university authorities and 0 otherwise); *x*_9_= *x*_10_= gender of faculty member, measured as a dummy (1 for male and 0 for female); *x*_11_= teaching area, measured as a dummy (1 for teaching in the natural sciences and 0 for teaching in the social sciences); *x*_12_= awareness of CBT, measured as a dummy (1 for faculty member knows of CBT and 0 otherwise); *x*_13_=teaching load/week, measured in number of teaching hours per week; and *x*_14_= number of undergraduate students/class. *ε*_*i*_ is the error term.

## Results and discussion

### Socioeconomic characteristics of faculty members

Tables 1 and 2 present the socioeconomic characteristics of the respondents. The results showed that most of the sampled faculty members of Kwame Nkrumah University of Science and Technology (KNUST) (80%) were males. Also, there was no significant difference in the percentages of males who were willing to adopt CBT and those unwilling (Table 3). The dominance of males is consistent with Mtebe and Raisamo (2014) who found similar results for lecturers in Tanzania. With an average age of 42.3 years, the age distribution showed that at least 55% of teaching staff of the university were between the ages of 41 to 60 years (Tables 1 and 2). Also, no significant difference was found between the ages of faculty members willing to adopt the methodology and those not willing (Table 3). With a greater majority of teaching staff (54.7%) being lecturers, very few (8.3%) were found to be professors. With the KNUST being a science and technology university, it was not surprising that most of the sampled respondents (76.7%) were in the natural sciences. That notwithstanding, there was no significant difference in the proportion of those in the natural sciences who were potential adopters and non-adopters (Table 3). Generally, most respondents (84%) were of the view that there were no incentives in adopting CBT as a teaching strategy notwithstanding its numerous advantages. This result corroborates a similar finding reported by Malufu et al. (2016) that sampled lecturers at universities in Bulawayo of Zimbabwe lacked institutional support for adoption of teaching technologies. Meanwhile, the results revealed a significant difference between potential adopters and non-adopters who opined that incentives were given for adopting CBT (Table 3). The implication is that those who were willing to adopt CBT were those who received incentives for extra duties undertaken in the university. Also, 80.3% of them stated that teaching aids needed for successful adoption of CBT were unavailable but this is inconsistent with Kumar and Daniel (2016) who reported huge respondents’ endorsement of availability of teaching resources in Fijian Polytechnic Institutions. A greater percentage of the respondents (88%) were also of the opinion that generally, supervision in the university was very effective. Table 3 also showed that potential adopters of CBT were those who experienced effective supervision of their duties in the university. This indicates the importance of supervision in the implementation of teaching innovations. Moreover, awareness of CBT was not a problem as 72.3% of sampled faculty knew of the methodology. This reflects the results in Table 3 that there was no significant difference between potential adopters and non-adopters who were aware of CBT. As desired, majority of the sampled respondents (64.7%) stated that they were willing to adopt CBT as a teaching methodology. This however disagrees with the findings of Acakpovi and Nutassey (2015) that found poor readiness of polytechnic staff to implementation of CBT. Table 2 also showed that, generally, faculty members of the KNUST have 12.6 years of teaching experience. This is good news for the university given the aforementioned average age of faculty. Generally, there was no significant difference in the teaching experience of potential adopters and non-adopters (Table 3). The average teaching load recorded by respondents per week was 8.2 h. This is within range and could enhance adoption of CBT as lecturers are expected to teach 12 h a week. This is supported by the results in Table 3 that the teaching loads of potential adopters were significantly lower than those unwilling to adopt the methodology. It is also interesting to note that 42.7% of the sampled faculty members stated they engaged in one or more off-campus activities such as teaching in other universities on part-time basis and participating in media discussions, while the remaining 57.3% remarked they were glued to work on campus. The number of faculty members engaged in off-campus activities is quite high, and this could prevent them from making ample time for teaching activities on campus. Given that CBT approach uses more time than traditional teaching approaches, the net result is that faculty members will likely be reluctant at adopting CBT in their teaching activities. It is also interesting and worrying to note that only 28.7% of the sampled faculty members stated they have participated in a CBT workshop before. This has the potential to prevent faculty members from adopting the methodology since obviously, most of them may not know what CBT is all about. Table 3 presents a significant difference in the percentages of potential adopters and non-adopters who have participated in one or more CBT workshop(s), indicating that potential adopters were those who ever participated in a CBT workshop. Also, for those who have ever participated in CBT workshops, only 11.6% attended competency-specific workshops. Competency-specific workshops are desired as they are targeted at specific fields or teaching areas that give participants in-depth knowledge of appropriate CBT approaches for specific topics in the field. Finally, the total number of undergraduate students per class was 184 (Table 2) and this was significantly lower for potential adopters of CBT vis-à-vis non-adopters (Table 3). The total number of students per class (184) is quite high if CBT is to be adopted since students must be close to lecturers to be able to see and learn employable skills. With such high numbers, most students sit in the class room and find it difficult seeing what really goes on, making implementation of CBT difficult.

**Table 1:** Socioeconomic characteristics of respondents

Variable	Sub level	Frequency	Percentage
Gender	Male	240	80
	Female	60	20
	Total	300	100
Age in years	≤ 40	80	26.7
	41–60	170	56.7
	≥ 60	50	16.6
	Total	300	100
Rank	Professors	25	8.3
	Senior lecturers	65	21.7
	Lecturers	164	54.7
	Assistant lecturers	46	15.3
	Total	300	100
Teaching area	Natural science	230	76.7
	Social science	70	23.3
	Total	300	100
Availability of incentives	Yes	48	16
	No	252	84
	Total	300	100
Availability of teaching aids	Yes	59	19.7
	No	241	80.3
	Total	300	100
Effective supervision	Yes	264	88
	No	54	18
	Total	300	100
Awareness of CBT	Yes	217	72.3
	No	83	27.7
	Total	300	100
Off-campus activities	Yes	128	42.7
	No	172	57.3
	Total	300	100
Participation in CBT workshop	Yes	86	28.7
	No	214	71.3
	Total	300	100
Participation in competency-specific training	Yes	10	11.6
	No	76	88.4
	Total	86	100
Willingness to adopt CBT	Yes	194	64.7
	No	106	35.3
	Total	300	100

Source: Survey, 2017

**Table 2:** Descriptive statistics of respondents’ characteristics

Variable	Min	Max	M	SD
Age of respondent	31	68	42.3	8.5
Teaching experience	1	40	12.6	9.3
Teaching load/week (hours)	6	15	8.2	6.4
Undergraduate students/class	20	581	184	20.6

Source: Survey, 2017

**Table 3:** Test of equality of means of characteristics of adopters and non-adopters of CBT

Variable	Willing to adopt CBT	Unwilling to adopt CBT	Difference	*t* value
Age of respondent	44.5	43.4	1.10	1.67
Teaching experience	13.7	11.9	1.80	0.88
Teaching load	6.43	14.1	− 7.67	− 11.4**
Undergraduate students/class	25.3	174	− 148.7	− 12.45***
Male gender	69.4	65.5	3.94	1.81
Teaching area	0.54	0.31	0.23	0.67
Availability of incentives	0.79	0.31	0.48	7.84*
Availability of teaching aids	0.81	0.21	0.60	3.52**
Effective supervision	0.64	0.24	0.40	6.14***
Awareness of CBT	0.71	0.64	0.07	0.79
Off-campus activities	0.63	0.68	− 0.05	0.29
Participation in CBT workshop	0.73	0.19	0.54	5.44***
Participation in competency-specific training	0.81	0.67	0.14	0.94

Source: Survey, 2017

Note: The asterisks indicate levels of significance: ***significant at 1%, **significant at 5%, and *significant at 10%

### Faculty’s perception on competency-based training

Table 4 presents the perception of faculty members on competency-based training. The percentage of respondents that strongly agreed, agreed, neutral, disagreed, or strongly disagreed are shown in parenthesis. For example, out of the 300 faculty members that participated in the survey, 31.7% strongly agreed that CBT makes students technically efficient, 40.3% agreed, 20.7% were indifferent, 3.3% disagreed, and 4% strongly disagreed. With 46% of the respondents strongly agreeing that CBT makes students innovative and creative, 33.3% agreed. The results also showed that whereas 41.7% strongly agreed that CBT gave students problem-solving skills, 36.7 agreed and just 4% and 3.7% disagreed and strongly disagreed, respectively. Generally, CBT was found to give students excellent communication skills since 32.7% of sampled faculty strongly agreed and 29.3% agreed. Most faculty members (36.3% strongly agreed and 32.3% agreed) believed that CBT could instill in students the ability to work in teams, making them team players. Table 4 also showed that faculty members are of the opinion that students’ interpersonal skills could be improved if CBT is adopted (41.7% strongly agreed and 35% agreed). With only 19% of the respondents strongly agreeing that CBT could boost students’ confidence, 32.7% agreed, 36.3 were indifferent, and very few disagreed (7.3%) and strongly disagreed (4.7%). The sampled faculty members had a positive perception of CBT being able to give students knowledge of ICT skills for work. Specifically, 36.3% of sampled faculty strongly agreed to the statement, 32.7% agreed, and only 8.3% of them opposed it. Over 50% of respondents (55.7%) agreed to the statement that CBT gives students flexibility and adaptability in seeking alternative employment in a changing world. Also, 51% of sampled faculty of the KNUST strongly agreed that CBT helps students become more reflective, self-directed, and capable of maintaining family and community relationships. Added to this is the positive perception that CBT gives students the ability to transfer skills to practical situations. This is true since 30.7% and 33.3% of the respondents strongly agreed and agreed, respectively. Moreover, 52.7% of the sampled faculty members of the university were of the opinion that CBT gave students the ability to network in a variety of situations. Furthermore, with 27.7% of the respondents strongly agreeing to the statement that CBT helps students to organize and express ideas clearly, 38% agreed and very few of them (2%) objected to it. For students to be able to work methodically, 30.3% of the respondents strongly agreed that CBT was key, 41.3% agreed, and very few (6.3%) were of the opposite view. Finally, 33% and 35.3% of sampled faculty members of KNUST strongly agreed and agreed respectively to the statement that in CBT, employable skills form integral part of the design and structure of study programs, assessments, and staff development. The perception index of 0.49 implies that generally sampled faculty members of the KNUST had a positive perception of the potential of CBT in instilling in students employable skills. The index further implies that, generally faculty members agreed to the perception statements in Table 4. This is good news for the KNUST especially as the university strives to position herself to put quality graduates on the job market through emphasizing the use of CBT in teaching at the university. The above results are in consonance with similar findings obtained by Shao and Bruening (2005) that teachers supported curriculum reforms and they were interested in trying new ideas in their teaching practice. Also, the results are in agreement with van der Vleuten (2015) that competency-based education promotes relevant employable skills.

**Table 4:** Faculty’s perception on competences-based student-centered approach

Statements	Strongly agree (1)	Agree (0.5)	Neutral (0)	Disagree (− 0.5)	Strongly disagree (− 1)	Mean score
CBT makes students technically proficient	95 (31.7)	121 (40.3)	62 (20.7)	10 (3.3)	12 (4.0)	0.46
CBT makes students innovative and creative	138 (46.0)	100 (33.3)	58 (19.3)	3 (1.0)	1 (0.3)	0.62
CBT gives students problem-solving skills	125 (41.7)	110 (36.7)	42 (14.0)	12 (4.0)	11 (3.7)	0.54
CBT gives students excellent communication skills	98 (32.7)	88 (29.3)	52 (17.3)	35 (11.7)	27 (9.0)	0.32
CBT makes students team players	109 (36.3)	97 (32.3)	45 (15.0)	33 (11.0)	16 (5.3)	0.42
CBT gives students interpersonal skills for work	125 (41.7)	105 (35.0)	59 (19.7)	6 (2.0)	5 (1.7)	0.57
CBT gives students self-confidence	57 (19.0)	98 (32.7)	109 (36.3)	22 (7.3)	14 (4.7)	0.27
CBT gives students knowledge of ICT skills for work	109 (36.3)	98 (32.7)	68 (22.7)	10 (3.3)	15 (5.0)	0.46
CBT gives students flexibility and adaptability in seeking alternative employment in a changing world	73 (24.3)	167 (55.7)	54 (18.0)	4 (1.3)	2 (0.7)	0.51
CBT gives students logical and critical thinking skills	139 (46.3)	109 (36.3)	47 (15.7)	2 (0.7)	3 (1.0)	0.63
CBT helps students become more reflective, self-directed, and capable of maintaining family and community relationships	153 (51.0)	107 (35.7)	35 (11.7)	3 (1.0)	2 (0.7)	0.68
CBT gives students knowledge of sociocultural demands at work	132 (44.0)	112 (37.3)	52 (17.3)	2 (0.7)	2 (0.7)	0.62
CBT gives students the ability to transfer skills to practical situations	92 (30.7)	100 (33.3)	80 (26.7)	16 (5.3)	12 (4.0)	0.41
CBT gives students the ability to network in a variety of situations	98 (32.7)	158 (52.7)	38 (12.7)	2 (0.7)	4 (1.3)	0.57
CBT helps students to organize and express ideas clearly	83 (27.7)	114 (38.0)	74 (24.7)	18 (6.0)	11 (3.7)	0.4
CBT gives students the ability to work methodically	91 (30.3)	124 (41.3)	66 (22.0)	10 (3.3)	9 (3.0)	0.46
In CBT, employable skills form integral part of the design and structure of study programs, assessments, and staff development	99 (33.0)	106 (35.3)	60 (20.0)	26 (8.7)	9 (3.0)	0.43
Perception index						0.49

Source: Survey, 2017

### Determinants of adoption of competency-based training by academics in Ghana

Table 5 presents the maximum likelihood estimates of the logistic model for factors influencing adoption of CBT by the sampled faculty members of KNUST. From the results, a likelihood ratio (LR) statistic of 119.34 with a chi-squared (chi^2^) distribution at 14 degree of freedom was highly significant at 1% level. This means that at least one of the explanatory variables in the model has a significant effect on faculty members’ decision to adopt CBT and that the explanatory variables jointly influence faculty members’ decision to adopt CBT. The Pseudo *R*^2^ of 0.369 is small and is expected, especially with binary choice models.

**Table 5:** Logistic regression coefficients and marginal effects of factors influencing adoption of CBT

Variable	Coefficient	Std. err	*z*-statistic	Marginal effect	*P* value
Constant	− 1.425	0.987	− 1.44		0.149
Age of respondent	1.524	1.296	1.18	0.160	0.240
Teaching experience	0.062	0.871	0.07	0.007	0.943
Off-campus activities	− 0.032	0.423	− 0.08	− 0.003	0.940
Competency-specific training	0.009	0.074	0.13	0.001	0.900
Participation in CBT workshop	1.593***	0.514	3.1	0.167	0.002
Effective supervision	2.765***	0.431	6.41	0.290	0.000
Availability of teaching aids	1.692***	0.566	2.99	0.177	0.003
Availability of incentives	0.875**	0.426	2.06	0.092	0.040
Support from current curricula	0.01	0.513	0.02	0.001	0.985
Gender	0.001	0.017	0.07	0.013	0.943
Teaching area	− 0.017	0.039	− 0.44	− 0.002	0.657
Awareness of CBT	0.034	0.039	0.86	0.004	0.392
Teaching load/week	− 0.065***	0.02	− 3.19	− 0.007	0.001
Undergraduate students/class	− 0.001**	0.987	− 1.44	− 0.002	0.035
Number of observations	300				
Log likelihood	− 102.113				
LR chi^2^ (14)	119.34				
Prob >chi^2^	0.000				
Pseudo *R*^2^	0.369				

Source: Survey, 2017

Note: The asterisks indicate levels of significance: ***significant at 1% and **significant at 5%

The study found adoption of CBT as a teaching strategy by the sampled faculty members to be influenced by a number of factors. The main factors which were found to be significant were participation in CBT workshops, effective supervision, availability of teaching aids, availability of incentives, teaching load per week, and number of undergraduate students per class (Table 5).

The coefficient and the marginal effect of participation in CBT workshops were positive and significant for adoption of CBT (*p* < 0.01). The marginal effect revealed that participation in CBT workshops would result in a 16.7% increase in the probability of the faculty member adopting CBT as a teaching strategy (Table 5). The implication is that faculty members who have attended CBT workshops before were more likely to adopt the methodology in their teaching activities. This is because, at these workshops, faculty members become abreast of what really goes into successful implementation of the methodology. Also, the workshops bring to light the agent need for faculty members to strive to join the crusade for use of CBT in teaching in tertiary institutions in order to be able to inculcate into their students employable skills. This result agrees with empirical results obtained by Shao and Bruening (2005), Balash et al. (2011), and Koster et al. (2017) who emphasized effective training of faculty members as a major factor influencing adoption of teaching technologies by faculty members.

The results showed that effective supervision of teaching activities of faculty members of the university was statistically significant at the 1% significance level for adoption of CBT. The marginal effect implies that effective supervision of faculty members’ teaching activities by university authorities would increase the likelihood of faculty members’ adoption of CBT by 29% (Table 5). This is because, once implemented, effective supervision will let faculty members follow required CBT procedures, thereby increasing adoption. This agrees with similar findings reported by Jarvis (2009).

The coefficient of the variable representing availability of teaching aids or resources for use of CBT had a positive and significant effect on adoption of CBT (*p* < 0.01). The marginal effect revealed that availability of teaching aids would increase the probability of a faculty member adopting CBT by 17.7% (Table 5). This is because a decision to use CBT in teaching should be complemented by certain teaching and learning materials, and therefore, when these materials are not present, faculty members are discouraged from employing it. This result agrees with that of Shao and Bruening (2005), Iddrisu et al. (2014), and Kirwa (2016) that lack of effective laboratory tools and other resources is a major cause of low patronage of CBT by the staff of tertiary institutions. That is, laboratory facilities that are required to enhance practical training have been progressively degrading, forcing facilitators to make their teaching more theoretical than practical (Acakpovi and Nutassey 2015).

The coefficient and the marginal effect of availability of incentives were positive and significant for adoption of CBT (*p* < 0.05). The marginal effect revealed that availability of incentives would result in a 9.2% increase in the likelihood of faculty members adopting CBT (Table 5). This is because adoption of CBT, which is relatively time consuming, means that faculty members may have to leave some of their off-campus income-generating activities, and therefore, with increased incentives, adoption of CBT becomes attractive. This finding corroborates those of Nyarko (2011) as well as Balash et al. (2011) that reported poor incentives as a major impediment to the implementation of CBT.

The results also showed that the variable for teaching load per week was negative and statistically significant at the 1% level for adoption of CBT. The marginal effect implied that an increase in teaching load would decrease the probability of a faculty member adopting CBT by 0.7% (Table 5). Given that adoption of CBT is time consuming, an increase in teaching load will likely encourage the use of lecture approaches that is not student-centered where the lecturer will have to hurriedly teach a class to make way for another.

Finally, the coefficient of the variable representing number of undergraduate students per class had a negative and significant effect on adoption of CBT (*p* < 0.05). The marginal effect revealed that an increase in the number of undergraduate students per lecture hall would decrease the likelihood of faculty members’ adoption of CBT by 0.2% (Table 5). This is because facilities that complement adoption of CBT methodologies accommodate up to a particular number of students and when that number is exceeded, lecturers have no choice than to resort to non-student-centered lecture approaches. This result is in line with that of Iddrisu et al. (2014) that high enrolment is one of the challenges that hamper the successful implementation of CBT in tertiary institutions.

## Conclusions

This study examined the perception and adoption of competency-based training (CBT) by academics in Ghana using primary data from Kwame Nkrumah University of Science and Technology (KNUST), Kumasi, Ghana. The study concludes that generally faculty members of KNUST had a positive perception of the potential of CBT in instilling in students employable skills. The main factors which were found to be significant in influencing or determining adoption of CBT were participation in CBT workshops, effective supervision, availability of teaching aids, availability of incentives, teaching load per week, and number of undergraduate students per class.

The implication is that faculty members who have attended CBT workshops before were more likely to adopt the methodology in their teaching activities. This is because, at these workshops, faculty members become abreast of what really goes into successful implementation of the methodology. Also, the workshops bring to light the agent need for faculty members to strive to join the crusade for use of CBT in teaching in tertiary institutions in order to be able to inculcate into their students employable skills. Once implemented, effective supervision will let faculty members follow required CBT procedures appropriately, thereby increasing adoption. Availability of teaching aids is also important for adoption of CBT because a decision to use CBT in teaching should be complemented by certain teaching and learning materials, and therefore, when these materials are not present, faculty members are discouraged from employing it. Incentives will also increase adoption of CBT because adoption of CBT, which is relatively time consuming, means that faculty members may have to leave some of their off-campus income-generating activities, and therefore, with increased incentives, adoption of CBT becomes attractive. Moreover, given that adoption of CBT is time consuming, an increase in teaching load will likely encourage use of lecture approaches that is not student-centered where the lecturer will have to hurriedly teach a class to make way for another. Finally, an increase in the number of undergraduate students per lecture hall will impede adoption of CBT because facilities that complement adoption of CBT methodologies accommodate up to a particular number of students, and when that number is exceeded, lecturers have no choice than to resort to non-student-centered lecture approaches.

By way of recommendation, provision of appropriate teaching and learning resources that complement adoption of CBT, incentives, and competency-based education training for academics by university authorities and stakeholders in Ghana’s tertiary education will enhance the adoption of competency-based education methodologies. This can be done through close collaboration between academia and industry/practitioners aimed at providing the needed resources for implementation of CBT and also practitioners contributing to the development of curricula for CBT syllabi. Academics are also encouraged to be very innovative in their choice of teaching strategies notwithstanding the challenges they face in their quest to employ CBT approaches in their teaching activities. The study also recommends that university authorities of KNUST should keep up with and add to their supervisory roles as this has proven to be effective in making faculty members adopt CBT in their teaching activities. Faculty members occupying key positions in the university are therefore encouraged to strengthen their supervisory roles if the benefits of CBT approaches are to be realized. In addition, for CBT to be given the attention it deserves by faculty members, there is the need to as much as possible cut-down on the teaching loads of faculty members. This is because, when teaching loads are reduced, faculty members can spend more time with students and this will let lecturers put in students, employable skills which will make them competitive in the job market after graduation. Added to this is the fact that, for teaching loads to be reduced, there is the need for university authorities to recruit new faculty members. Furthermore, for CBT to be duly implemented, there is the need for university authorities to cut-down on the number of undergraduate students per lecture hall. This will improve lecturer-student interaction and consequently inculcating into students, employable skills. Finally, a point of consideration deemed worthy of mentioning is the qualifications of faculty to facilitate the development of employable skills. What this means to universities as it relates to faculty having the right blend of academic and technical expertise to deliver quality CBT programs requires further investigation by future researchers.

## Abbreviations

CBT: Competency-based training
COTVET: Council for Technical and Vocational Education and Training
GEA: Ghana Employers Association
GoG: Government of Ghana
KNUST: Kwame Nkrumah University of Science and Technology
LR: Likelihood ratio
NCTVET: National Council on Technical and Vocational Education and Training
OECD: Organization for Economic Co-operation and Development
PI: Perception index
UNESCO: United Nations Educational, Scientific and Cultural Organization
